# Buckwheat (*Fagopyrum spp.*): Building Healthy Diets and Resilient Agrifood Systems

**DOI:** 10.1021/acs.jafc.5c17847

**Published:** 2026-04-24

**Authors:** Maria Eugenia Araujo Silva Oliveira, Hugo José Martins Carvalho, Cristina Martinez-Villaluenga, Cristina Yoshie Takeiti, Maria Teresa Pedrosa Silva Clerici

**Affiliations:** † Departamento de Ciência de Alimentos e Nutrição, Faculdade de Engenharia de Alimentos, Universidade Estadual de Campinas, Campinas, SP 13083-862, Brasil; ‡ Institute of Food Science, 593682 Technology and Nutrition (ICTAN-CSIC), Jose Antonio Novais 6, Madrid 28040, Spain; § Embrapa Agroindústria de Alimentos, Avenida das Américas, 29501, Rio de Janeiro, Rio de Janeiro 23020-470, Brasil

**Keywords:** pseudocereal, phenolic compounds, slowly digestible starches, glycaemic index, protein-interactions

## Abstract

Buckwheat (*Fagopyrum* spp.), mainly represented by common buckwheat (*Fagopyrum esculentum*) and Tartary buckwheat (*Fagopyrum tataricum*), is a climate-resilient pseudocereal with high nutritional value. Its unique composition, rich in proteins, slowly digestible starches, and phenolic compounds, links sustainability with health-promoting properties. The aim of this review was to reinforce and summarize current knowledge on buckwheat as a source of bioactive compounds, the molecular mechanisms underlying starch interactions, technological processing, and its application in gluten-free products. Evidence indicates that buckwheat proteins and phenolic compounds interact with starch, modulating its digestibility and contributing to improved glycemic control. Advances in food technology, particularly through 3D printing and steam explosion treatments, offer opportunities to design gluten-free foods with a low glycemic index (GI) and desirable sensory attributes. These developments position buckwheat as a promising ingredient for celiac and nonceliac consumers, as well as for individuals managing glucose metabolism or using GLP1 analog medications.

## Graphical abstract

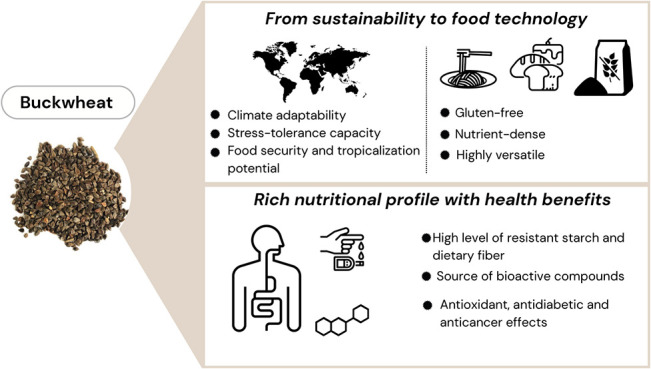

## 1 Introduction

Global hunger has risen sharply since 2019 and remains persistently high. In 2023, nearly 1 in 11 people worldwide faced hunger, while over 2 billion experienced moderate to severe food insecurity; this is 383 million more than before the COVID-19 pandemic.^1^ Although the share of countries reporting high food prices declined from 60% in 2022 to 50% in 2023, this remains more than three times the prepandemic level. These alarming levels of global hunger, food insecurity, and malnutrition are driven by conflict, climate variability, and economic instability, further compounded by inequality and limited access to nutritious food.^1^

The war in Ukraine, one of the world’s major wheat exporters, amplified by multiple extreme weather events, marked a second major global shock to food markets, disrupting trade routes, amplifying uncertainty, and reinforcing inflationary pressures set in motion by the pandemic.^2^ Simultaneously, climate change continues to intensify, with rising temperatures, irregular precipitation, and surface water flows projected to become more variable over most land regions within seasons and from year to year. The increasing frequency of extreme weather events impacts agricultural productivity, water availability, and ecosystem balance.^3^

Under these conditions, agricultural systems must adapt through the diversification of crops that are resilient, nutritionally valuable, and environmentally sustainable. In this context, buckwheat (*Fagopyrum esculentum* spp.), a pseudocereal belonging to the Polygonaceae family, stands out as a promising candidate. Compared with conventional cereals, it exhibits great agronomical advantages: short growing cycle (∼90 days), drought resistance, ability to grow in nutrient-poor soils, beneficial interactions with pollinators, low fertilizer requirements, good biomass accumulation, and water-use efficiency.^4–7^ These characteristics make buckwheat suitable for marginal land and sustainable crop rotation systems.

Buckwheat originates from Central Asia and was transferred by nomadic populations to Central and Eastern Europe. In the 13th century, it reached some importance in Germany, Austria, and Italy, although it later declined with the expansion of modern cereal crops.^8^ Today, two varieties of buckwheat are commonly cultivated: common buckwheat (*Fagopyrum esculentum*) and tartary buckwheat (*Fagopyrum tataricum*).^9^ In 2023, global production of buckwheat was 2.2 million tons across 2.18 million ha with Europe contributing nearly half (48.9%), followed by Asia (44.8%), the Americas (5.8%), and Africa (0.4%)^10^ (Figure 1).

**Figure 1:**
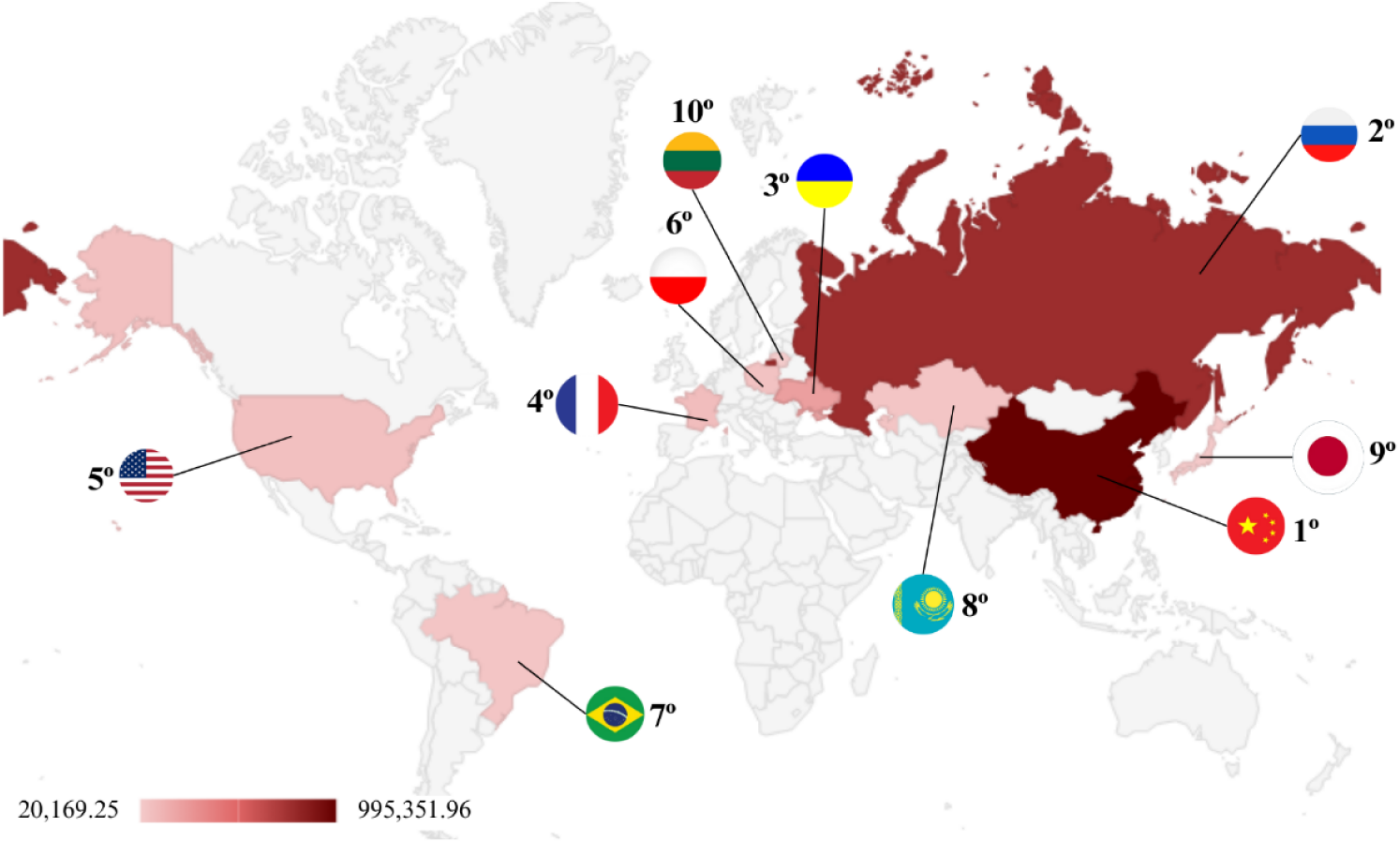
Production quantities by region and top 10 producers of buckwheat in the world between 1994 and 2023. (Source: FAOSTAT, 2023).

Global production of coarse grains is projected to reach 330 Mt by 2034, up by around 33 Mt from the base period. African countries are expected to contribute 45% of this increase, driven by strong demand growth stemming from rapid population expansion and continued reliance on staple foods, which in turn incentivize higher local production. On a country basis, India (+4.1 Mt), Ethiopia (+3.2 Mt), and Nigeria (+2.7 Mt) will contribute the most.^11^ However, the diversification of underutilized crops such as buckwheat remains essential to strengthen food system resilience and nutritional security because future agricultural land expansion is projected to be limited due to urbanization and the implementation of environmental and sustainability policies.

Although still underutilized, buckwheat represents a valuable alternative to conventional grains due to its nutritional and functional properties. It provides high-quality proteins, bioactive phenolic compounds, and slowly digestible starches, making it particularly relevant for individuals with celiac disease, gluten sensitivity, diabetes, or those using GLP-1 analog medications.

Despite these advantages, buckwheat remains underutilized at the global level. This underutilization is likely driven by multiple, interconnected factors, including its limited incorporation into mainstream food products, sensory and technological challenges in some applications, variability in processing performance, and the dominance of established cereal supply chains. In addition, the health and functional advantages of buckwheat have not always been effectively translated into consumer-oriented products with consistent quality and broad market acceptance. These constraints help explain why buckwheat, despite its strong agronomic, nutritional, and functional potential, still occupies a relatively marginal position in many food systems (Figure 2).

**Figure 2:**
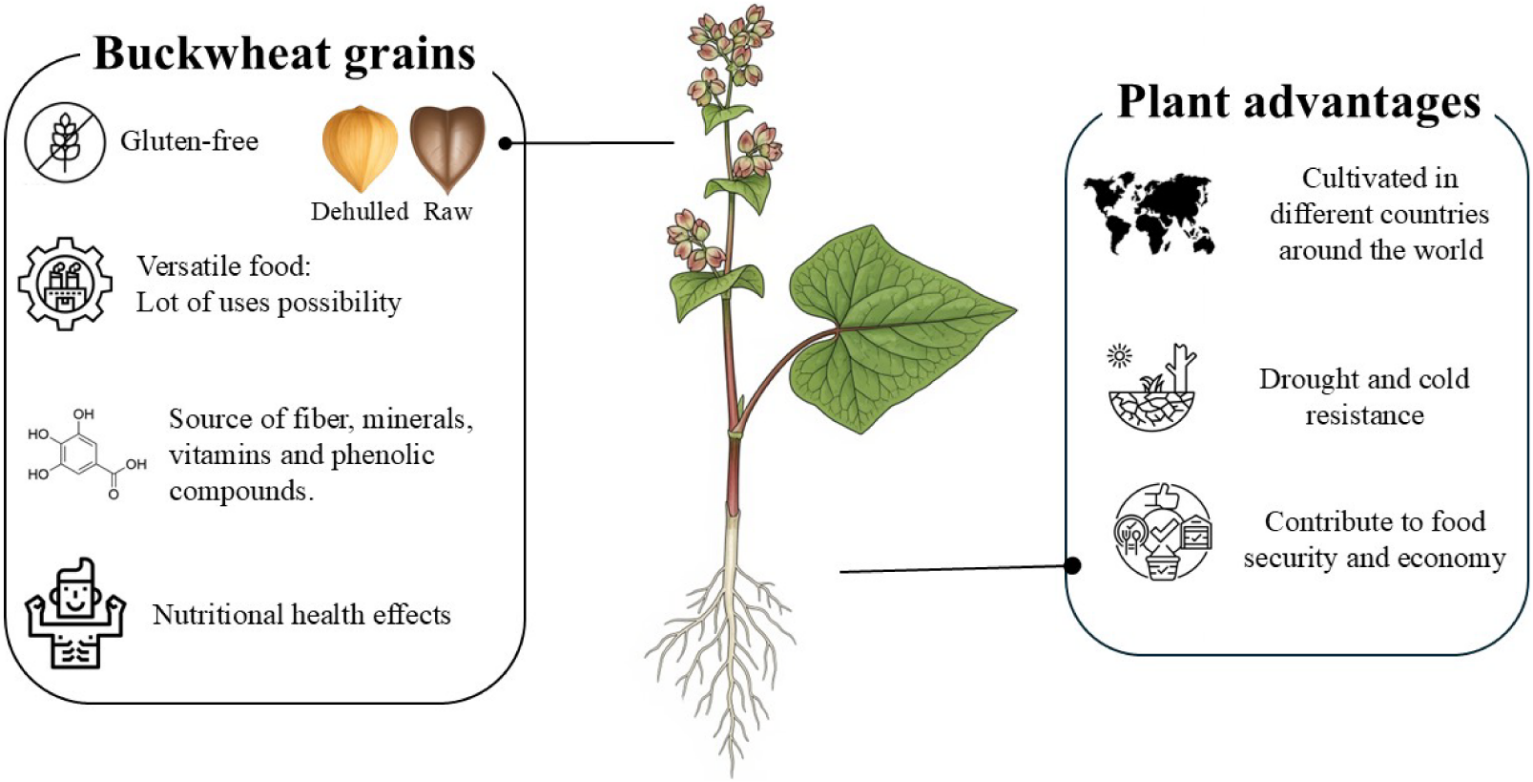
Overview of principal advantages of buckwheat plants and grains.

Recent reviews have underscored buckwheat’s agronomic, nutritional, and socio-economic relevance, emphasizing its potential role in climate-resilient food systems.^12–14^ However, these works have primarily focused on compositional and genomic aspects, with limited attention to the molecular interactions between starch, proteins, lipids, and phenolic compounds, which critically determine buckwheat’s digestibility, glycemic response, and functional performance in gluten-free foods. Moreover, the impact of technological processes, such as extrusion, germination, and fermentation, on starch structure and bioaccessibility remains underexplored, despite their relevance to designing health-oriented products with improved sensory and nutritional attributes.

In this context, the present review provides an updated synthesis of recent advances linking starch–macromolecule interactions, digestibility, and processing functionality in buckwheat-based systems. By integrating biochemical, nutritional, and technological perspectives, this work highlights how food processing can be harnessed to develop gluten-free, low-glycemic products that combine sustainability, functionality, and consumer appeal. This integrated approach aims to bridge the gap between molecular understanding and product innovation, positioning buckwheat as a model ingredient for the next generation of climate-resilient and health-promoting foods.

## 2 Data Search Strategy

The studies that were included in this narrative review were found in the Web of Science, ScholarGoogle, and PubMed databases, complemented by information from organizational sites and private global market research sources. The search covered publications from 1997 to 2026. Keywords included “processes”, “pseudocereal”, “buckwheat”, “*Fagopyrum escutelum*”, “*Fagopyrum tataricum*”, “common buckwheat”, “Tartary buckwheat”, “phenolic compounds”, “bioactive compounds”, “products”, “health benefits”, and “cancer” as well as their combinations using the Boolean operator “AND”. To complement the scientific literature, commercially available food and beverage products made with buckwheat were identified on online retail platforms (Amazon and iHerb) up to March 2026 (Table 1). These data were used to illustrate current market applications and product diversification trends.

**Table 1:** Commercially Available Buckwheat Products^a^

**Category**	**Brand**	**Product**	**Country**
**Grain**	Eden	Organic Whole Grain	USA
	Bob’s Red Mill	Organic creamy hot cereal	
	Lil Bucks	Sprouted crunch	
		Sprouted seeds	
	Food to live	Grain	
	Makfa	Buckwheat groats	Russia
**Flour**	Amisa	Buckwheat flour	UK
	Giroil		Brazil
	Kodilar		
	Coopernatural	Organic flour	
	Vero Nuttri		
	Bob’s Red Mill		USA
		Pancake and Waffle Mix	
	Arrowhead Mills	Organic pancake and waffle mix	
	New Hope Mills	Pancake mix	
**Pasta**	Eden	Organic pasta	
	Mezzani	Soba	Brazil
	Ibogawa		Japan
	AOI Foods		
	Amisa	Spaghetti	UK
**Snack**	Maine Crisp Company	Crakers	USA
**Granola**	Clusterbucks	Granola clusters	
**Drink**	Nature’s own factory	Tea	UEA
**Crispbread**	Amisa	Organic gluten-free	UK
	Le pain des fleurs		France

a Note: Searches were carried out on Web sites (Amazon and iHerb) until 2026, March.

## 3 Buckwheat Macromolecules and Phenolic Profile

### 3.1 Starch

Buckwheat is an important primary source of carbohydrates, and starch represents the main reserve carbohydrate, varying between 65% and 78% of the grain’s dry weight in its composition. This value is higher than that observed in other pseudocereals and traditional cereals such as rice and wheat. The endosperm concentrates most of the starch (70% to 80%), which is composed of 72–80% amylopectin and 19–28% amylose, reaching 35% depending on the variety and cultivation conditions.^15^ The wet-milling process has commonly been applied to concentrate the starch, using chemicals such as NaHSO_3_ (0.2%) to facilitate the removal of nonstarch components.^16^

According to Gao et al.,^17^ 12 varieties of Buckwheat starch granules display considerable morphological diversity, with spherical, irregular, or polygonal shapes and smooth surfaces, typically with a size distribution ranging from 3 to 20 μm in diameter. This result is related by Zhu^16^ that reports starch granule variation ranging from 2 to 15 μm.

Regarding the crystalline structure of buckwheat starch, a typical A-type can be observed, with diffraction peaks around 15° and 23° 2θ, similar to the pattern presented by other pseudocereal and cereal starches.^18^ Besides, buckwheat starch can present the amount of extra-long amylopectin unit chains (DP > 100),^19^ which may confer unique pasting and gel texture properties of technological interest, as illustrated by Gao et al.^17^ These authors reported that rheological properties, according to RVA analysis, reveal a low peak viscosity (601–862 mPa s), low pasting temperature (66–71 °C), and higher final viscosity (100–1537 mPa·s), indicating a strong tendency toward retrogradation and gel stability.^17^

In addition, buckwheat is notable for its high resistant starch in native form, averaging 18–23% and reaching 30% in some cultivars. This contributes to its low glycemic index and metabolic benefits, including improved glucose homeostasis and gut health, although its content may vary depending on the thermal processing applied, such as autoclaving, cooking, or boiling, which decreases the resistant starch content, as demonstrated by Lu and Baik,^20^ where cooked buckwheat groats showed 1.6–3.8 g/100 g. These data confirm that RS can be mostly classified as RS1, meaning that raw starch loses strength after cooking.

The digestibility of buckwheat starch is further modulated by its interactions with other grain components. Zhu^16^ reported that buckwheat flour-based products have a low glycemic index, which was attributed to interactions between lipids (short-chain fatty acids), fibers, proteins, and bioactive compounds such as gallic acid and quercetin, which can decrease the enzyme susceptibility of starch, thereby slowing hydrolysis and attenuating postprandial glycemic responses. These structural and compositional features position buckwheat as a valuable carbohydrate source for developing low-glycemic foods with enhanced physiological functionality.

### 3.2 Proteins

Buckwheat varieties (*F. esculentum* and *F. tataricum*) show protein contents of 6.82–15.02%, higher than other conventional cereal grains such as rice (6.50–12.40%) or wheat (11.0–12.8%). The protein fraction is composed mainly of globulins (43.3–64.5% of total proteins), followed by albumins (12.5–18.2% of total proteins), prolamins (0.8–2.9% of total proteins), and glutelins (8–22.7% of total proteins).^21^

It is important to highlight the absence of low molecular weight prolamins (30 kDa), which are responsible for celiac disease, reinforcing their use in gluten-free formulations. Both buckwheat varieties demonstrate a well-balanced amino acid profile, which can vary depending on the cultivar, origin, and growing conditions. The presence of lysine, with a content above 6%, leads to the potential of supplement grain-based diets, since it is a limiting factor in conventional cereals such as corn and wheat. Furthermore, it has a high presence of arginine (between 10 and 11%), glutamic acid (23 to 25%), and aspartic acid (10 to 11%). This composition enhances the nutritional quality of buckwheat proteins and suggests potential metabolic benefits, including improved nitrogen balance and modulation of lipid and glucose metabolism. Compared to cereals such as rice and corn, buckwheat also stands out for having higher sulfur amino acids such as cysteine and methionine.^22^

### 3.3 Lipids

The lipid content of buckwheat is relatively low (1.2 to 5.4%), yet this fraction is highly important in the context of its physiological and technological roles due to its favorable fatty acid composition, with approximately 80% of total lipids being unsaturated fatty acids, predominantly concentrated in the embryo. Oleic (35–44%) and linoleic acids (31–41%) are the major constituents, with higher levels of oleic in *F. esculentum* and linoleic in *F. tartaricum*.^23^

This predominance of monounsaturated and polyunsaturated fatty acids, together with the low proportion of saturated ones, mainly palmitic and stearic acids, confers a health-promoting lipid profile. These fatty acids are associated with cardioprotective effects, antiinflammatory action, and hypocholesterolemic effects, contributing to the reduction in the risk of metabolic and cardiovascular diseases.^24,25^

From a technological perspective, the oleic/linoleic ratio varies between species and genotypes, influencing oxidative stability, shelf life, and the sensory attributes of processed products. Thus, the combination of high-quality proteins and a favorable lipid profile reinforces buckwheat’s potential as a promising alternative to conventional cereals, particularly in the formulation of gluten-free and health-oriented foods.^26^

### 3.4 Phenolic Compounds

Buckwheat (*F. esculentum* and *F. tataricum*) is recognized as an important source of bioactive compounds, which, in addition to basic nutrients, contribute to health benefits. Its high levels of phenolic compounds (TPC) stand out particularly in the tartary variety, whose values can exceed 1100 mg GAE/100 g, of which approximately 180 compounds have already been detected and identified^27^ (Figure 3).

**Figure 3:**
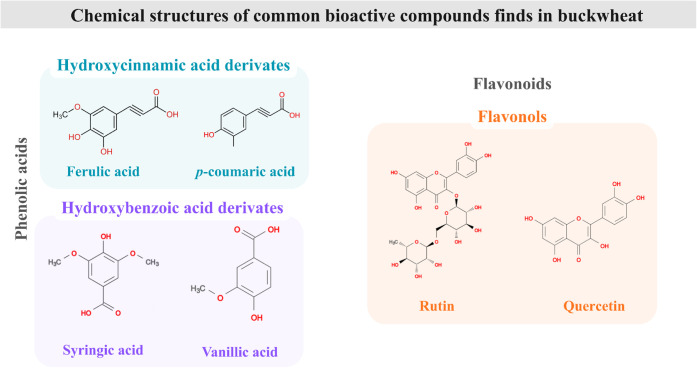
Major bioactive compounds of buckwheat.

Many of the polyphenols present in buckwheat can be found mostly in free form (>90% of the total), conferring high bioavailability, with emphasis on phenolic acids such as ferulic, coumaric, syringic, and vanillic acids. Among the flavonoids, rutin is the main compound, with much higher concentrations in the tartary variety (2.78 to 18.67 mg/g) compared to the common variety, and is predominantly found in the pericarp. Phenolic acids, although in lower concentrations, contribute to the antioxidant capacity, complementing the effects of flavonoids. The tartary variety also has a higher total flavonoid content (from 4.91 to 22.74 QE mg/g) and more expressive antioxidant activity. These values reflect not only genetic differences but also the strong influence of agronomic and environmental factors, such as cultivar, geographic origin, growing conditions, sowing date, and planting density.^26^

From a functional perspective of buckwheat, the high concentration of phenolic compounds provides antioxidant, antiinflammatory, antihypertensive, and hypocholesterolemic properties, as well as a potential contribution to reducing the risk of colon cancer.^27^ However, the processing method can strongly influence its composition. Therefore, maximizing the functional benefits of buckwheat requires not only appropriate variety selection and agronomic management but also processing strategies that preserve its rich bioactive profile.

## 4 Food Application of Buckwheat in the Gluten-Free Market

The number of consumers purchasing gluten-free products is increasing steadily worldwide over the past decade, driven not only by the increasing prevalence of celiac disease and nonceliac gluten sensitivity but also by a broader consumer perception of gluten-free foods as healthier and more natural alternatives. Gluten-free consumers tend to be highly ingredient-conscious, preparing their own meals, preferring a short list of ingredients, and showing interest in buying and trying out new products.^28–30^ Naturally gluten-free, buckwheat represents an attractive ingredient for this expanding market. It combines technological versatility with a favorable nutritional profile, rich in high-quality proteins, slowly digestible starch, and bioactive compounds, which allows its incorporation into a wide range of gluten-free products. Buckwheat-based products can already be found in several countries, especially in Europe, where they appear in different formats such as grains, flour, snacks, pancake and waffle mixes, pasta, and beverages (Table 1).

Buckwheat flour is a key product and can be used alone or in combination with other pseudocereal or cereal flours to improve texture, color, and sensory quality. Its application has deep roots in traditional recipes across various cuisines, such as Japanese soba noodles and Russian blinis 
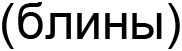
, pancakes traditionally served with side dishes like sour cream, caviar, or smoked salmon. In recent years, research has explored the application of buckwheat in baked foods, extruded snacks, and fermented beverages, highlighting its potential to enhance the nutritional and functional properties of gluten-free formulations (Table 2).

**Table 2:** Effect of Different Processes on Buckwheat and Their Products in the Last 5 Years

Product	Buckwheat type	Process	Major findings	References
Bread (chia + buckwheat)	-	Premix	The premixes made by buckwheat and chia flour exhibited higher protein, ash, crude fiber and polyunsaturated fatty acid content and antioxidant activity than the commercial premix and its bread (commercial sample).	^36^
Noodles	-	Cold extrusion	Buckwheat noodles presented high levels of potassium, phosphorus, magnesium, caffeic acid, 4-hydroxybenzoic acid, catechin and rutin which have high antioxidant activity	^37^
Bread	-	Baking	The breads made with refined buckwheat flour had good specific volume, lower firmness during storage, greater protein digestibility, and better sensory acceptance when compared to the formulations made with 30% and 45% of whole buckwheat flour	^38^
Polenta (Buckwheat + corn)	Common and Tartary	Milling	Tartary buckwheat polentas were darker, firmer and had a higher intensity of bitter taste and astringency than those prepared with common buckwheat.	^39^
			The addition of 30% and 40% of buckwheat flour had no differences between the species.	
Biscuits	Common	Fermentation	Fermentation improved the nutritional quality of buckwheat flours.	^40^
			High bioaccessibility of melanoidins after *in vitro* digestion.	
Flour	Tartary	Extrusion cooking	Decrease of relative crystallinity, endothermic enthalpy, slowly digested starch, and resistant starch content.	^41^
			Increase of amylose molecular weight of branched starches, degree of gelatinization, water absorption index, starch digestibility, rapidly digested starch content, and glycemic index.	
Biscuits	-	Cooking	Buckwheat flour increased the high-water absorption capacity (139%), water solubility (1.50%), foam capacity (15.47%), and stability (89.21%) compared to almond four in gluten-free biscuits	^42^
Bread	Common	Baking	Buckwheat hull had little effect on loaf volume and crumb firmness of bread but decreased specific volume and baking loss.	^43^
			The particle-size (tissue-scale: 500–100 μm and cell-scale: 50–10 μm) influence amylopectin retrogradation and starch recrystallization during bread storage, being higher in tissue-scale.	
Couscous	Common	Germination	Germination process (72 h) increased the content of phenolic compounds (rutin and quercetin) and antioxidant activity upon cooking.	^44^
			The addition of 50% of germinated buckwheat flour improved texture characteristics (in terms of cohesiveness, gumminess, and resilience) and enhanced sensory attributes (reduced astringency, bitter taste, and aftertaste).	
Bread	Common (Kora Variety)	Microwave	Microwave increased the consistency and elastic response of dough compared to control (80% rice flour +20% corn starch) and native buckwheat flour.	^45^
			Breads made by flour treated with microwave processes had a significant reduction in glucose release during *in vitro* starch digestion and an increase in protein digestibility.	
Cookies	Tartary	Stem explosion	Stem explosion enhanced the water-extractable arabinoxylans content by 24–152% and decreased the α-helix content, starch, and relative crystallinity in flour.	^46^
			Cookies made by flour treated by SE had lower digestibility of starch and higher amount of resistant starch.	
Biscuits	Tartary	3D printing	Buckwheat provided high-quality printing results	^47^
Flour (mixed flour made by teff, pearl millet, and buckwheat)	-	Milling	Blending pearl millet and buckwheat flour into teff flour resulted in nutrient-enhanced composite flour with improved functional and pasting properties.	^48^
Processed grain	Tartary	Boiling, steaming, deep-frying, stir-frying, and popping	Whole seeds (Tartary buckwheat) cooked preserves rutin and prevents its degradation into quercetin rather than flour. Boiling method (100 °C/10 min) did not substantially alter the rutin and quercetin levels.	^49^
			Slow heating (25 °C) water may cause partial degradation of rutin, whereas using preboiled water for cooking can effectively prevent such degradation.	
			Reduction in rutin content following deep-frying and steaming without a corresponding increase in quercetin levels	
			Popping process can result in rutin loss	

Beyond consumer health motivations, sustainability considerations increasingly shape purchasing behavior. With the global population set to exceed 8.6 billion by 2030, consumers want to make more responsible choices, driving the demand for green products. Consumers do not see the need to transform current agricultural practices but want to understand regenerative agriculture better. Young people (especially Gen Z) are environmentally conscious and concerned about the future of the planet.^31^ In this scenario, buckwheat has emerged as a sustainable and interesting crop. This crop may have the potential to be an alternative to wheat in the future, as it has good biomass accumulation and water-use efficiency under extreme drought conditions.^6^

As discussed earlier, buckwheat is an important food due to its versatility, is rich in fiber and demonstrates beneficial effects on glucose *in vitro* ^32^ and *in vivo* ^33^ assays. Another alternative for the inclusion of buckwheat-based products is for people using antiobesity medications such as Ozempic, Wegovy, Mounjaro, Zepbound, and similar glucagon-like peptide-1 (GLP-1) drugs. The growing use of GLP-1 analogues for obesity and metabolic disorder management, commonly termed the “Ozempic effect”, is reshaping consumer behavior and creating new challenges for the food industry. Users of these medications often experience reduced appetite and lower food intake, increasing the demand for foods that deliver high nutrient density, balanced macronutrient profiles, and satiety-enhancing properties.^34^

In this context, nutrient-dense and functionally rich foods are expected to gain greater prominence, with innovation focusing on formulations that optimize protein, fiber, and micronutrient delivery in smaller portions.^35^ Buckwheat fits this paradigm particularly well due to its balanced amino acid profile, slowly digestible starch, and high soluble fiber content, which support glycemic control, satiety, and gut health. Therefore, the incorporation of buckwheat into next-generation “food-as-medicine” formulations represents not only an opportunity for innovation but also a strategic response to evolving nutritional and metabolic demands.

## 5 Starch Digestibility and Health-Promoting Properties of Buckwheat

### 5.1 Effects of Processing on Starch Digestibility and Glycemic Control

Diabetes is a serious, chronic disease that occurs either because the pancreas does not produce enough insulin (a hormone that regulates blood sugar, or glucose), or because the body cannot effectively use the insulin produced.^50^ As mentioned earlier, starch is the main component of Tartary and common buckwheat (approximately 70%). Many foods rich in starch can induce a rise in glucose and affect the glycemic and insulin response.^51^ The *in vitro* starch digestion has been divided into three categories: (i) rapidly digestible starch (RDS); (ii) slowly digestible starch (SDS); and (iii) resistant starch (RS).^52^

The digestibility and enzymatic susceptibility of starch can also be affected by internal and external factors. The main factors are (i) the starch modification by processing; (ii) chemical modifications, (iii) the interactions between starch and protein, fat, polysaccharides, and phenolic compounds; and (iv) starch source, granule size, amylose content, and crystalline structure.^53,54^

Diverse processing methods are used to modify the structural and physicochemical properties, and consequently the starch digestibility of buckwheat. These changes depend on the type of processing, botanical source, and particle size. Buckwheat fine flours (<250 μm particle size) contain more protein, starch, riboflavin, low phytic acid content, and inhibited dipeptidil peptidase IV (DPP-IV) compared to coarse flours (>250 μm particle size).^55^

High hydrostatic pressure treatment facilitates complex formation through hydrogen bonding and hydrophobic interactions, and heat treatment induces starch despiralization, thereby increasing the binding sites available for flavonoids of tartary buckwheat.^56^ Dense structure impedes the diffusion of enzymes and substrate interactions during the digestive process. Heat treatment reduced the porosity of the buckwheat noodles. A strong positive correlation was observed between porosity and both estimated glycemic index and digestion rate constant *k*_1_, suggesting that enhanced pore formation improves enzymatic accessibility and accelerates starch hydrolysis.^57^

Gaseous ozone treatment (0, 2.5, 7.5, 15, and 20 min) increases the detectable carbonyl and carboxyl group content, improves the amylose content, amorphous region, and resistant starch, but decreases the crystalline layer and rapidly digested starch.^58^ On the other hand, extrusion leads to starch becoming more accessible, exposing interior regions that are more vulnerable to enzymatic attack and facilitating starch hydrolysis, thereby decreasing the SDS, the relative crystallinity, and increasing the glycemic index.^59^

Xiao et al.^54^ investigated the modification of starch and flour of native tartary buckwheat modified by heat-moisture treatment with different moisture levels (200, 250, 300, and 350 g/kg). This process increased the onset, peak and conclusion temperatures, SDS, and RS, while decreasing the pasting viscosities, enthalpy, and gel hardness. These authors attributed these changes to the high content of lipids, proteins, and nonstarch polysaccharides in tartary buckwheat flour, as well as the intra- and intermolecular reactions.

Du et al.^60^ studied the *in vitro* digestibility of buckwheat cultivars (eight common and eight tartary varieties) in comparison to wheat (eight samples). Buckwheat starches showed lower content of amylose (3–4%) than wheat starch, but no significant difference in the digestibility of tartary and common buckwheat compared with that of wheat was noted. These findings demonstrated that digestibility is not related to botanical sources, although it may be more influenced by starch structure.

Extrusion cooking causes the degradation of amylopectin and amylose and alters the microstructure of tartary buckwheat starch, probably due to high temperature and shearing, increasing the lipid–amylose or polyphenol–amylose interactions. This is assumed to be the basis for the V-type conformation and self-aggregation of lipids and/or polyphenols.^61^ Altering extrusion conditions with high moisture induced interactions between tartary buckwheat protein (TBP) and tartary buckwheat starch (TBS), causing the inhibition of amylopectin degradation and promotion of amylose disruption during extrusion. Moreover, TBP could interact with TBS through hydrogen bonds and the Maillard reaction. The addition of TBP (4–20%) increased the starch short-range and long-range ordered structures, which was probably attributed to the accelerated starch retrogradation since extrusion induced protein–starch interaction and protein aggregation.^62^

### 5.2 Interactions between Buckwheat Starch and Phenolic Compounds

Starch and phenolic compounds interact through noncovalent interactions, forming inclusion complexes in the form of amylose single helices facilitated by the hydrophobic effect, or complex with much weaker binding, mostly through hydrogen bonds.^62^ According to Zhou et al.^56^ the interaction between flavonoids and starch is predominantly mediated by hydrogen bonding, with flavonoids being bound to starch through surface adsorption or shallow spiral cavity encapsulation. Rutin and quercetin can impact the viscosity and texture profile of tartary buckwheat, but the strength of these interactions remains unclear. Some authors have suggested that the iodine-binding capacity of starch can be taken as an indicator of the strength of these interactions^63^ and rutinose (rutin disaccharide groups) can restrict the binding of hydrogen between the benzene ring and starch molecules.^64^

In addition to changes in the physicochemical properties of starch, phenolic compounds can form complexes with starch, in which the complexation can protect phenolic compounds from degradation and isomerization in the stomach and improve their bioaccessibility.^65^ Flavonoids (rutin, quercetin, and kaempferol) have strong antioxidant and α-glucosidase inhibition activities, as well as relatively weak α-amylase inhibition activity *in vitro*.^66^

Quercetin had a stronger inhibitory effect on *in vitro* digestion of tartary buckwheat starches than rutin, in which quercetin can significantly change the structure of enzymatic molecules, thereby inhibiting enzyme activity.^67^ Quercetin can impact the *in vitro* starch digestibility, change the physicochemical properties, and act as physical barriers between enzymes and starch of tartary buckwheat. These impacts are probably due to two mechanisms: (i) the inhibition of α-amylase activity by quercetin in a competitive manner and α-glucosidase activity in a mixed-type manner, and (ii) the interaction between quercetin and starch, thus indirectly affecting digestion.^68,69^

Rutin interacted with enzymes mainly by C–H and O–H groups on the glycoside structure, which induced steric hindrance and restricted the inhibitory effect of the quercetin fraction. The glycoside structure weakened inhibition of rutin on digestive enzymes in free forms rather than influencing its antidigestive effects in bound forms with starch.^32^

Zhou et al.^70^ investigated the inhibitory activities of quercetin on α-glucosidase and α-amylase in both *in vitro* and *in vivo* assays. In this study, quercetin showed very strong inhibitory activity on α-glucosidase, where quercetin acts as a noncompetitive inhibitor of α-glucosidase. According to the α-amylase inhibition activity, the effect was far weaker than that of acarbose. Quercetin improved postprandial hyperglycemia and reduced blood glucose.^71^

### 5.3 Interactions between Buckwheat Starch and Macromolecules

Interactions between starch and macromolecules can hinder enzyme access to starch, which impacts starch digestion as they limit the access of amylolytic enzymes, effectively decreasing their bioactivity and bioavailability. The formation of ternary complexes (starch-lipid–protein complexes) is influenced by various factors such as (i) pH, (ii) fatty acid chain length, (iii) amylose polymerization degree, and (iv) temperature. The combination of these factors leads to the resistant starch formation^72^ The term “resistant starch” was used by Englyst in the 1980s and can be defined as “starch made resistant to α-amylase digestion”.^73^ Several studies in scientific literature have already identified the beneficial effects of resistant starch, such as (i) enhancing short-chain fatty acids (SCFAs); (ii) improving satiety and reducing energy intake; (iii) reducing inflammation, and (iv) improving insulin sensitivity.^74–76^

Proteins limit the initial hydrolysis of starch by wrapping around starch granules and inhibiting the movement of water during gelatinization, and fats combine with amylose to form starch-fat complexes that limit the hydrolysis rate of starch, causing changes in the thermal properties of buckwheat starch.^77,78^ In general, buckwheat lipids are fatty acids predominated by unsaturated acids C_18:2_ and C_18:1_ and can affect various properties of buckwheat starch.^79,80^ The presence of these lipids has a significant impact on starch digestibility since (i) they may interact with the hydrophobic helical structure of amylose; (ii) inhibit enzymatic digestion by forming starch-lipid or fatty acid complexes; and (iii) contribute to the suppression of the amylose-iodine complex formation and the inhibition of pancreatin.^78,81,82^

### 5.4 AntiInflammatory, Antioxidant and Antidiabetic Activities

A growing body of evidence supports that buckwheat and its derived products exert multiple biological effects relevant to metabolic health, including antioxidant, antiinflammatory, antiadipogenic, and antidiabetic activities. These bioactivities are largely attributed to the synergistic effects of phenolic compounds (notably rutin and quercetin), d-chiro-inositol, resistant starch, and bioactive peptides formed during bioprocessing.^83–85^ Figure 4 summarizes the main cellular, animal, and human trials that have examined these effects.

**Figure 4:**
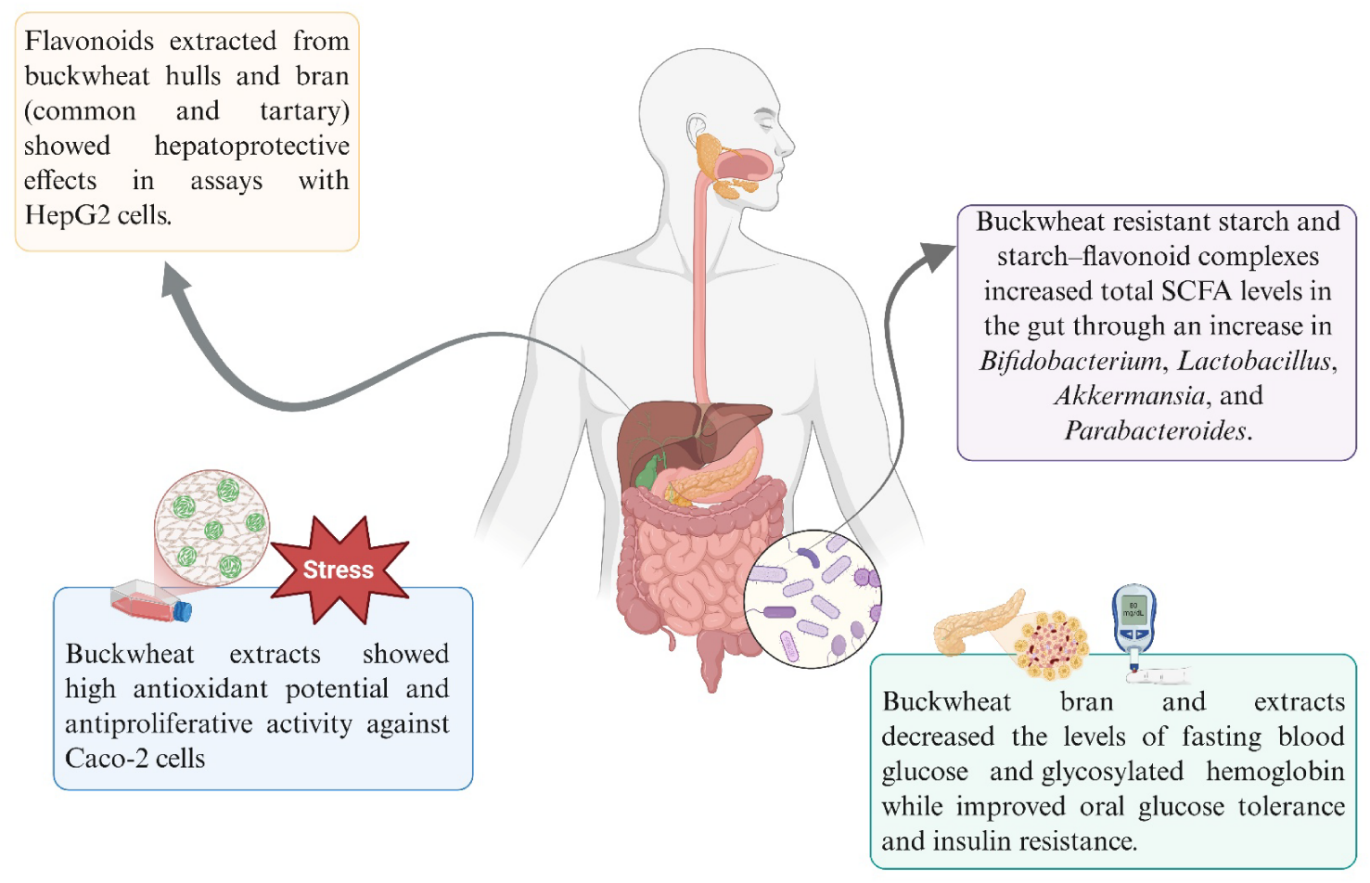
Cellular, animal, and human health benefits of buckwheat.

The most cited benefits were about antioxidant and antiinflammatory effects, especially for rutin, which significantly inhibited Th2 signals in T cells, showing exacerbated antiallergy activity,^84^ hepatoprotective and antidiabetic effects from d-chiro-inositol^85^ and prebiotic effect, in which buckwheat-resistant starch increases the population of bacteria in the gut.^86^

Regarding the animal models and clinical trials, the most cited benefits were the antidiabetic effects. *In vitro* assays showed that buckwheat had antiadipogenic activity, which inhibited the formation of fat droplets using a C3H10T1/2 mesenchymal stem cell line—a conversion of a preadipocyte cell to a mature lipid-containing adipocyte.^87^ Sprouted tartary buckwheat methanolic extract exhibited higher antiinflammatory potential and anticancer effects in lipopolysaccharide-stimulated macrophage (RAW264.7) cells and lung (H1975) cells.^88^

### 5.5 Cellular Evidence

At the cellular level, extracts from buckwheat hulls and bran, rich in rutin, vitexin, isoorientin, and hyperoside, exerted strong antioxidant and cytoprotective effects on hepatocytes (HepG2) and intestinal epithelial cells (Caco-2), reducing H_2_O_2_- and high-glucose-induced oxidative damage.^89,90^ These studies demonstrated restoration of mitochondrial membrane potential, increased antioxidant enzyme activity (superoxide dismutase, catalase, and glutathione-*S*-transferase), and enhanced cellular antioxidant capacity.

Recent investigations using HepG2 models also showed that polyphenolic fractions from tartary buckwheat downregulated HMG-CoA reductase and upregulated cholesterol 7α-hydroxylase (CYP7A1), indicating improved cholesterol catabolism and a potential hypolipidemic effect.^91^ Furthermore, a buckwheat-derived peptide (Ala-Phe-Tyr-Arg-Trp, AFYRW) enhanced cellular glucose uptake in insulin-resistant hepatocytes, suggesting a novel insulin-sensitizing mechanism associated with peptide bioactivity.^92^

Together, these studies indicate that buckwheat bioactives can modulate key metabolic pathways related to oxidative balance, lipid homeostasis, and glucose metabolism, providing a mechanistic basis for their antidiabetic potential. However, most of these findings derive from isolated extracts or pure compounds, without considering digestion, bioaccessibility, or interaction within complex food matrices—an important limitation for translational relevance.

### 5.6 Animal Studies

Animal models further corroborate the metabolic benefits of buckwheat-derived compounds. In diabetic KK-Ay mice, fagopyritols from tartary buckwheat activated the PI3K/AKT insulin-signaling pathway, resulting in improved glycemic control.^93^ Similarly, supplementation with **s**prouted tartary buckwheat (2.5–5%) in high-fat diet-fed mice reduced body weight gain, hepatic lipid accumulation, and insulin resistance, while improving serum lipid profiles.^94^

Steam explosion-modified tartary buckwheat bran showed **r**emarkable antidiabetic and gut-modulating effects in db/db diabetic mice, evidenced by lower fasting glucose, glycated hemoglobin, and liver damage, alongside increased short-chain fatty acids (SCFAs) and beneficial shifts in gut microbiota composition, notably higher *Bifidobacterium*, *Alloprevotella*, and *Ruminococcaceae* populations.^95^ These microbial changes were associated with increased expression of intestinal GLP-1 and GPR43 receptors, suggesting a gut–metabolic axis mechanism mediated by buckwheat-resistant starch.

Moreover, ethanolic extracts of fermented tartary buckwheat improved glucose tolerance, hepatic glycogen storage, and antioxidant defense (SOD and CAT activity) in Kunming mice.^96^ Consistent results were observed in recent studies, where starch–flavonoid complexes prepared by high hydrostatic pressure restored intestinal dysbiosis in diabetic mice, decreasing *Bacteroidetes* and increasing *Lactobacillus* and *Bifidobacterium* relative abundance, which contributed to blood glucose regulation.^97^

Intervention with buckwheat resistant starch also prevented weight gain and increased SCFA levels in high-fat diet mice, confirming the prebiotic and metabolic benefits of buckwheat starch fractions.^86^ Collectively, these animal studies demonstrate that both native and processed buckwheat fractions can modulate insulin signaling, oxidative stress, and gut microbiota, thereby contributing to improved metabolic outcomes.

### 5.7 Clinical Studies

Clinical trials, though limited in number, provide supportive evidence that buckwheat consumption can ameliorate metabolic markers in humans. In a randomized crossover study involving individuals with type 2 diabetes, consumption of buckwheat crackers modulated incretin hormones (GLP-1, GIP) and pancreatic polypeptide levels, reflecting improved glycemic regulation.^98^

A larger crossover intervention with twenty-one adults consuming buckwheat-based pasta, bread, crackers, and biscuits for 8 weeks reported significant reductions in total cholesterol (−4.7%), LDL cholesterol (−8.5%), triglycerides (−15%), fasting glucose (−5.8%), and insulin (−17%), independent of demographic variables.^99^ Moreover, in individuals with both type 1 diabetes and celiac disease, fiber-enriched buckwheat pasta induced a more stable postprandial glucose response compared to conventional pasta, supporting its use in gluten-free diets aimed at glycemic control.^100^

While these human trials corroborate the metabolic benefits observed in experimental models, most have short durations and lack standardized *in vitro* digestion or kinetic analyses (e.g., INFOGEST protocol) to correlate starch and protein digestibility parameters with glycemic outcomes. This represents an important research gap for future investigations, particularly given the structural and compositional diversity of buckwheat starch and its complexes with phenolics and proteins.

## 6 Conclusion

Buckwheat is more than a gluten-free pseudocereal of high nutritional value; recent literature indicates that it is a structurally complex food matrix in which starch digestibility and metabolic functionality are strongly modulated by interactions with proteins, lipids, and phenolic compounds. A central insight emerging from this review is that the health-related performance of buckwheat cannot be explained by its composition alone, but its macromolecular organization is altered by processing and translated into digestibility, glycemic response, and product quality.

Recent studies show that processing is a decisive factor in directing these outcomes. Technologies such as extrusion, microwave treatment, germination, fermentation, and steam explosion can either increase starch accessibility and glycemic potential or, conversely, promote structural rearrangements and starch–macromolecule complexes that slow enzymatic hydrolysis and improve functional performance. This duality helps explain the variability reported across studies and highlights that the nutritional value of buckwheat-based foods depends not only on the raw material but also on the processing route and formulation strategy.

Another important conclusion is that Tartary and common buckwheat should not be viewed as interchangeable ingredients. Their differences in phenolic content, sensory profile, and response to processing create distinct technological and nutritional opportunities. Overall, buckwheat emerges as a promising platform for the development of climate-resilient, gluten-free, and metabolically relevant foods, but progress in the field will depend on moving from compositional description toward the mechanism-based design of ingredients and products.

## 7 Future Perspectives

Future progress in buckwheat research should move toward a more predictive and application-oriented framework, linking genotype, processing, structure, and physiological response. Addressing the underutilization of buckwheat will require coordinated efforts across breeding, processing, product development, and market positioning. Breeding programs should target not only agronomic performance but also traits relevant to food applications, such as starch structure, phenolic profile, and sensory acceptability. In parallel, processing technologies should be optimized to improve texture, palatability, and functional performance, while preserving or enhancing the bioactive compounds. Greater emphasis should also be placed on the development of consumer-acceptable buckwheat-based foods, including its use as a partial substitute for conventional flours in widely consumed products. Together with stronger integration between research, industrial innovation, and sustainability-driven food policies, these strategies could help move buckwheat from an underutilized crop toward a more prominent role in resilient and health-oriented food systems.

In addition, standardized experimental platforms are needed to generate evidence across studies. In particular, the application of harmonized in vitro digestion protocols, such as INFOGEST, combined with starch hydrolysis kinetics, glycemic response modeling, and bioaccessibility assessment of phenolics and peptides, would allow clearer interpretation of how structural changes induced by processing affect functionality. Future studies should also focus on mechanism-based elucidation of starch–protein–lipid–phenolic interactions in real food matrices rather than isolated fractions only. Multiomics and advanced structural tools, including metabolomics, microscopy, X-ray diffraction, FTIR, and molecular modeling, should be integrated to identify which supramolecular arrangements are most effective in reducing starch digestibility while preserving sensory quality. Finally, translational validation is essential. More human intervention studies are needed to confirm whether the structural and biochemical effects observed in vitro and in animal models translate into improved glycemic control, satiety, lipid metabolism, and gut health. These studies should use well-characterized buckwheat products with defined formulation and processing histories, allowing the establishment of evidence-based claims and clearer positioning of buckwheat in functional, gluten-free, and health-oriented markets.
